# Understanding the Challenges and Uncertainties of Seroprevalence Studies for SARS-CoV-2

**DOI:** 10.3390/ijerph18094640

**Published:** 2021-04-27

**Authors:** David McConnell, Conor Hickey, Norma Bargary, Lea Trela-Larsen, Cathal Walsh, Michael Barry, Roisin Adams

**Affiliations:** 1 National Centre for Pharmacoeconomics, St James’s Hospital, D08 HD53 Dublin, Ireland; hickeyc2@tcd.ie (C.H.); trela@stjames.ie (L.T.-L.); Cathal.Walsh@ul.ie (C.W.); mbarry@stjames.ie (M.B.); roadams@tcd.ie (R.A.); 2 Department of Pharmacology and Therapeutics, Trinity College Dublin, D08 HD53 Dublin, Ireland; 3 Health Research Institute and MACSI, University of Limerick, V94 T9PX Limerick, Ireland; Norma.Bargary@ul.ie

**Keywords:** COVID-19, SARS-CoV-2, coronavirus, seroprevalence, antibody testing

## Abstract

SARS-CoV-2 continues to widely circulate in populations globally. Underdetection is acknowledged and is problematic when attempting to capture the true prevalence. Seroprevalence studies, where blood samples from a population sample are tested for SARS-CoV-2 antibodies that react to the SARS-CoV-2 virus, are a common method for estimating the proportion of people previously infected with the virus in a given population. However, obtaining reliable estimates from seroprevalence studies is challenging for a number of reasons, and the uncertainty in the results is often overlooked by scientists, policy makers, and the media. This paper reviews the methodological issues that arise in designing these studies, and the main sources of uncertainty that affect the results. We discuss the choice of study population, recruitment of subjects, uncertainty surrounding the accuracy of antibody tests, and the relationship between antibodies and infection over time. Understanding these issues can help the reader to interpret and critically evaluate the results of seroprevalence studies.

## 1. Introduction

The SARS-CoV-2 virus is likely to be circulating in populations since December 2019 with the currently known first case recorded in Wuhan in China. Despite worldwide attempts at suppression, and in some countries eradication, the virus continues to circulate and in most countries it is unclear to what extent. However, many publications have indicated that it is circulating to a wider extent than the case incidence report [1,2,3].

Estimating the true number of people who have previously been infected with SARS-CoV-2 enables scientists and policy-makers to understand how the virus spreads in various settings, to retrospectively assess the performance of infectious disease models and hence improve future modeling and planning for further outbreaks, and to evaluate the effectiveness of restrictions aimed at curbing the spread of the virus. The quantification of those who have been exposed to infection is ideally done via direct testing for presence of the virus. Currently, naso and oropharyngeal swabs are the two main recommended upper respiratory tract specimen types for COVID-19 diagnostic testing, with detection of the virus being carried out via real-time reverse transcription-quantitative polymerase chain reaction (RT-PCR) [4,5,6]. However, identifying cases in this way is dependent on capturing those cases while a person is shedding virus.

An alternative method is to estimate the exposure of the virus via the presence of antibodies specific to SARS-CoV-2 in blood samples, which allow for a considerably longer detection period [6]. At a population level, antibody tests can be used to carry out “seroprevalence” studies, which estimate the proportion of people who have SARS-CoV-2 antibodies in their blood (as a proxy for previous infection). Seroprevalence studies have been the subject of much recent attention; however, estimates of total case numbers arising from these studies can be highly uncertain. Overlooking the uncertainties and limitations of these studies can lead to a flawed understanding of the disease and its spread, and potentially to poor policy decisions as a result. An example of this can be seen in the reaction to an early seroprevalence study in Santa Clara County California [7], which generated considerable controversy at the time, and was frequently cited when debating the potential need for nonpharmaceutical interventions (NPIs) against COVID-19 [8]. More recently, a study of blood donors in Manaus, Brazil, suggested an historic infection rate of 76% in the city by October 2020, above most estimates of a herd immunity threshold and therefore making future waves of infection extremely unlikely [9]. Despite this, a large outbreak unexpectedly followed shortly afterward in December 2020 and January 2021 [10].

These examples highlight the need for scientists and policy makers to critically evaluate seroprevalence studies and recognize their limitations. In this article, we therefore examine the main sources of uncertainty and common misunderstandings that can arise from such studies, and outline how their impact may be assessed. 

## 2. Background

### 2.1. The Problem with Confirmed Cases

**Figure 1:**
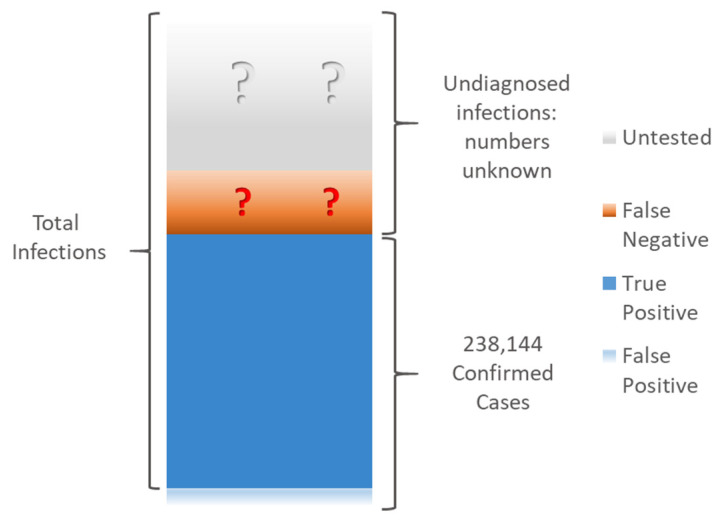
Total confirmed COVID-19 cases in Republic of Ireland as of 6 April 2021. Official COVID-19 case numbers (HPSC [11] 6 April 2021) are based on RT-PCR positive tests only (a small number of which may be false positives). The true number of infections also includes an unknown number of people who were either never tested, or else falsely tested negative.

As of 6 April 2021, there were a total of 238,144 confirmed cases of COVID-19 in Ireland [11]. As is the case in many (if not most) countries [2,12], there is good reason to believe that the true number of those who have been infected is considerably higher than official case numbers suggest, as outlined in Figure 1. Many of those who were infected were not tested at all, particularly in the earlier stages of the epidemic in Ireland, when testing was largely restricted to those showing two or more “typical” symptoms, healthcare workers, and high-risk groups. While testing is now more widespread, it is still likely that some mild or asymptomatic infections remain undiagnosed. Additionally, the RT-PCR tests used to detect active infection are not perfect [13]—according to one study [14], a currently infected person has at most a 67% chance of correctly testing positive (this figure can be much lower, depending on the length of time since exposure). This means that many infected people are recorded as testing negative and are not counted in the confirmed cases figures. While false positives (uninfected people testing positive) can also occur, these are much less common by comparison [15]. As a result, confirmed cases almost certainly underestimate total infection numbers to varying degrees around the world.

Underdetection and delayed reporting of (RT-PCR) confirmed COVID-19 cases is likely to have contributed to the wide ranges of predictions of future infections obtained from infectious disease models early in the pandemic [16]. The discrepancies between these predictions (sometimes arising from updating the same model with new data) lead to considerable confusion among policymakers, and may have eroded confidence in the usefulness of mathematical modeling in this context. Similarly, early estimates of case-fatality and case-hospitalization ratios varied considerably and were almost certainly affected by underdetection [17], potentially causing further confusion that influenced the public health response.

### 2.2. Seroprevalence Studies as A Solution

When infected with SARS-CoV-2, a person’s immune system produces antibodies to fight the virus, which, in the majority of cases, become detectable typically within 14 to 21 days of infection [18] and remain detectable for at least several months afterward [19,20]. There are a wide variety of test kits available for the detection of SARS-CoV-2 antibodies around the world [21], which work in different ways. Both point-of-care tests such as lateral flow immunoassays (LFIAs), and laboratory-based tests (e.g., enzyme linked immunosorbent assays, chemiluminescent assays) can be used, which may detect different types of antibody (IgA, IgG, IgM) or a combination thereof using whole blood, serum, or plasma samples. These tests may also differ in terms of the target SARS-CoV-2 antigen(s) (spike, membrane, or nucleocapsid proteins) used. By carrying out antibody tests on a group of people, we can determine who has been previously infected. These tests enable us to estimate seroprevalence in a population, that is, the proportion of people with SARS-CoV-2 antibodies in their blood, and thus estimate the total number of previous infections. A detailed introduction to some of the different study designs and antibody tests available, and how these may be chosen to align with the scientific question(s) being investigated, can be found in [22].

Many seroprevalence studies have been carried out around the world. While the results have varied considerably between studies and locations, they have consistently indicated that the true number of people previously infected is considerably higher than the official number of confirmed cases. For example, since June 2020 the Health Protection Surveillance Centre (HSPC) has been carrying out a seroprevalence study (SCOPI) in Ireland [23,24]. Preliminary results published in August 2020 estimated that 1.7% of the population aged 12–69 years in Ireland had previously been infected, corresponding to 59,500 total infections, which was approximately three times higher than the total number of confirmed cases in this age bracket at the time. Another example is the Prevalence of Antibodies to SARS-CoV-2 in Irish Healthcare Workers (PRECISE) study [25], designed to estimate seroprevalence among healthcare workers in two large Irish hospitals in October 2020 and again in March/April 2021. This study indicated that 39% of healthcare workers with detectable antibodies had not previously received a positive RT-PCR diagnosis, despite high rates of testing for active infection among these workers.

Studies such as these can provide useful information for researchers and policymakers in many ways. For example, comparing seroprevalence estimates in a broad population at various time points can give insight into the rate at which the disease has spread, which can in turn be used to assess the impact of nonpharmaceutical interventions (NPIs) during the given time period (e.g., [26,27]). Data from these studies can also be used to inform infectious disease models that aim to estimate the likely trajectory of the disease under various scenarios, and in turn to assess the impact of potential future NPIs accordingly. For example, seroprevalence studies have been used to estimate infection–fatality ratios (IFRs) [28,29], which can then be used in modeling studies to assess future policy decisions around NPIs or vaccination campaigns. Estimating seroprevalence among specific groups of interest (e.g., healthcare or other essential workers, residents of long-term care facilities, school-aged children) can provide further information about how the disease spreads in various settings and help to identify those that carry a high-risk of transmission, which can also inform policy decisions. For example, the PRECISE study indicated that workers in one hospital (St. James’s Hospital in Dublin) were approximately 3.7 times more likely to have evidence of previous infection compared with those in the other hospital studied (University Hospital Galway), even after accounting for differences in rates of contact with COVID-19 patients in the hospital, indicating that many healthcare workers are becoming infected in the community rather than in hospital settings. Other key insights from this study included the identification of healthcare assistants as having a high risk of infection, and also the elevated risk associated with living with others, particularly other healthcare workers. Similarly, seroprevalence studies among university students have been used to study risk factors for SARS-CoV-2 infection, such as international travel, living arrangements, contact with a confirmed COVID-19 case, and demographic factors [30].

With the rollout of mass vaccination campaigns, new applications of SARS-CoV-2 “serosurveillance” (that is, carrying our regular seroprevalence studies in a population) may arise. Indeed, as vaccinated individuals should also have detectable antibody levels, seroprevalence can provide a snapshot of the extent of COVID-19 immunity in the population at a given point in time, arising either from vaccination or from past infection [31]. While public health authorities will likely have reliable estimates of vaccine coverage in the population, there are concerns about infection- and vaccine-induced protection from COVID-19 declining over time [32,33], or about the prevalence of vaccine “nonresponders” who do not develop antibodies. As such, vaccine coverage in the population may not provide sufficient information about population immunity for public health authorities: carrying out routine serosurveillance in this setting could potentially address this gap. Therefore, as mass vaccination campaigns progress around the world, studies like these could provide invaluable information in jurisdictions aiming for herd immunity via vaccination in the future.

### 2.3. Uncertainty in Seroprevalence Studies

Unless we test everybody in the population, using a perfect (i.e., 100% accurate) test, we cannot calculate seroprevalence exactly: we can only estimate it alongside the associated uncertainty. When presented with the results of such a study, the reader will often need to assess the extent to which the true prevalence may differ from the estimate. Some of this uncertainty can be quantified, and this is usually presented in the form of “confidence” or “credible” intervals (discussed in Section 3.3) in the results. For example, the SCOPI study suggested that a range of 1.1% to 2.4% seroprevalence among 12–69 year olds in Ireland was plausible in August 2020, corresponding to between 39,800 and 85,200 infections in this age group. Confidence intervals like these capture some but not all of the uncertainty in the study results, and it can therefore be challenging for the nonexpert to make sense of these figures. There is a risk that many readers will naturally assume that all reasonable sources of uncertainty are accounted for by this range of values, and proceed under the assumption that the true number of past infections will almost certainly lie within this range. In practice, the uncertainty captured by interval estimates may be overshadowed by other sources of uncertainty that cannot be easily represented numerically. In what follows, we discuss some of the major sources of this uncertainty: issues around whom we include and exclude from the study, how we recruit participants, the role of random chance, and the limitations of antibody tests themselves. Understanding these issues can help to better assess the certainty of evidence provided by seroprevalence studies, and to avoid drawing misleading conclusions as a result.

## 3. Key Challenges in Seroprevalence Studies

In this section we discuss some of the main challenges that arise in estimating seroprevalence in a population, with a particular focus on how to interpret the results of studies and assess their limitations. These topics have been identified as a result of the Authors’ past experiences of communicating more general statistical and methodological uncertainty to scientists, clinicians and decision makers, together with a (nonsystematic) review of the issues arising in recent SARS-CoV-2 seroprevalence studies (particularly [7,9,24,25,27,34]).

### 3.1. Populations and Generalization—Seroprevalence Among Whom?

In a seroprevalence study, the group of people among whom we are trying to estimate prevalence of specific antibodies is called the population. Population could mean many things in this context: The Irish population as a whole, healthcare workers in Leinster, nursing home residents in Cork, etc. The study population is typically much larger than the group of people that we actually test, and care is needed to define it precisely: we may draw flawed conclusions from a study by generalizing the results to a population that is too different from those included in the study. For example, the SCOPI study enrolled people aged 12–69 [23]. It may be tempting to conclude that the prevalence of past SARS-CoV-2 infection in the over 70s will be similar to that in the study population; however, there are a number of reasons why this may not be the case. For example, the government’s cocooning advice may have reduced infection rates in this age group, while on the other hand, it is likely that hospital and nursing home outbreaks disproportionately affected the over 70s. Similarly, seroprevalence studies carried out in England [34], Scotland [35], the Netherlands [36], among others, use samples collected from blood donors. Many people with long-term medical conditions are not eligible to donate blood and thus would not be included. Moreover, even among those who are eligible to donate blood, those who actually do donate may differ in important ways from those who do not. Perhaps healthcare workers are more likely to donate blood, or maybe blood donors typically follow social distancing guidance more than the average person. It is not immediately obvious whether or not donors will be more or less likely to have been exposed than the general population. In the Manaus seroprevalence study mentioned in the Introduction, one possible explanation for the failure of (apparent) herd immunity to prevent a resurgence of cases is that using blood donors as a proxy for the wider population may have overestimated infection rates considerably [37].

The importance of limitations like these on the study population depends on how we intend to use the results. For example, given that the majority of COVID-19 deaths in Ireland occurred in the over 70s age group, it would be unwise to draw any firm conclusions about the overall infection fatality ratio (IFR) in Ireland from the SCOPI study (though it could be used to estimate the IFR among the under 70s). Indeed, failing to include high-risk groups in seroprevalence studies (such as nursing home residents, homeless individuals, or marginalized ethnic groups, for example) is a recognized challenge when attempting to estimate the IFR of the disease [29]. In general, it is essential for the reader to be aware of the population in which a seroprevalence has been carried out, as this is often more restrictive than we would like. While it may be case that seroprevalence is indeed similar in groups outside the study population (e.g., in the over 70s or among nonblood donors, respectively, in the two examples just discussed), drawing such a conclusion requires making assumptions, which represent an additional source of uncertainty that cannot be easily measured. The plausibility of these assumptions should be assessed using qualitative judgment and external information where possible.

### 3.2. Selecting Appropriate Samples

Typically, we cannot test everybody in a given population (except in cases where the population is small and easily identified, such as the residents of a specific nursing home). Instead, we select a sample of people to test, and based on the results, try to infer the prevalence in the population as a whole. However, we can only do this in a meaningful way if the sample is representative of the wider population, in terms of characteristics that affect the likelihood of having been previously infected.

**Figure 2:**
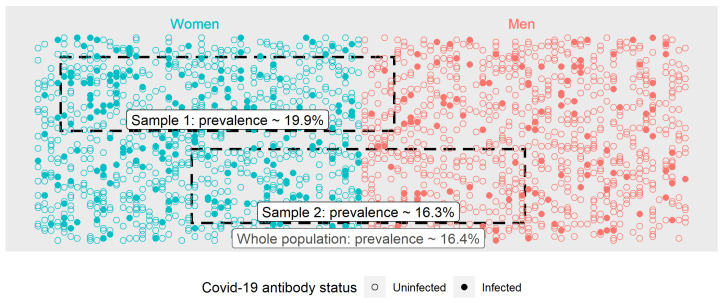
Importance of representative sampling. In this population, the rate of prior infection is higher among women. As a result, Sample 1, which is predominantly women, overestimates the prevalence of prior infection among the wider population. By contrast the prevalence in Sample 2, which contains approximately equal numbers of men and women, is much closer to that of the wider population.

As an example, imagine that we selected a sample of the population that was predominantly women (Sample 1 in Figure 2). While there may be no biological reason to suggest that either men or women are more or less likely to be infected, the prevalence of SARS-CoV-2 in a sample like this could differ from the general population for a number of reasons. For instance, it is quite plausible that workers in sectors that are predominantly women (e.g., healthcare) face a higher risk of being infected than the workforce at large. If these workers are over-represented in the sample, then the prevalence of SARS-CoV-2 may be overestimated by this study. By contrast, in a sample with approximately equal numbers of men and women (Sample 2 in Figure 2), this issue does not arise, and seroprevalence in the sample is much more likely to be close to the true (population) value.

There are many less obvious ways in which sample selection can introduce bias into prevalence estimates. For example, an early antibody study in Santa Clara, California [7] recruited participants using targeted Facebook ads. This could be a source of bias if Facebook users differ systematically from the population as a whole, and in ways that affect the likelihood of COVID-19 exposure; age profile, for example. Similar issues can arise in any study or survey in which participants self-enroll [38]. Other studies [39] have enrolled participants outside shopping centers. This approach can be better, provided that these areas cover a broad spectrum of the population in terms of age, sex, socioeconomic status, and other factors. When studies are carried out with the involvement of government agencies, it is often possible to select a representative sample of the population from an official “list” such as the electoral register or similar, and inviting those selected to participate. For example, the REACT study in England [27] selected participants at random from the National Health Service (NHS) patient list, which contains everybody registered with a general practitioner (GP) in England. Having an (almost) complete list like this from which to choose a sample is ideal, as it is much less likely to systematically exclude any large groups of the population, though this option is not always available to the study investigators.

Irrespective of who we invite to participate in a study, it will not necessarily be the case that all of those selected will agree to participate. This raises an important question—do the infection rates differ among those who agreed to participate and those who did not? For example, in some studies (e.g., [7,25] hereationsoflike these on the populationest both to those who use this information acknowledge their limitations) participants have been informed of their antibody test results afterward; it is possible that those who have recently experienced flu-like symptoms would be more likely to seek a test, compared with others in the population who had not. More generally, agreement to participate in a study could be influenced by a number of factors that in turn are related to the likelihood of previous exposure: socioeconomic status, trust in government or the medical profession, and many others. This issue is known as response bias, and is a common challenge that can influence the results of epidemiological studies [40,41,42].

Some of these challenges in sample recruitment can be accounted for in the study design, however, many cannot. In this case, the usual approach is to carry out an adjustment on the results, in order to reflect what would be obtained from a sample that “looks like” the population of interest in terms of predetermined characteristics (age, employment status, employment sector, living arrangements, and so on). There are a number of methods for doing this [43], and many well-designed studies will carry out some form of adjustment. However, this is not easy to do well and the reliability of the results depends on choosing appropriate factors to adjust [44,45]. Similarly, there are statistical methods available to adjust for bias due to nonresponse, though again care is needed to carry these out appropriately [46,47]. An example of sample adjustment can be found in the study [26], which selected numerous convenience samples of blood specimens that had been collected for other medical tests as part of routine clinical practice, and used them to estimate seroprevalence in each US state at various time points. The samples were adjusted (weighted) so that the resulting group of patients resembled the overall population of the corresponding US state, in terms of age, sex, and metropolitan status of their area of residence. While these factors likely explain some of the differences in exposure risk, there are potentially others that have not been accounted for—individuals who receive more frequent medical tests will still be over-represented in the sample, such as those with (multiple) underlying health conditions or those with better access to healthcare. Thus, while adjustment can address some of the issues with nonrepresentative samples, it is not a perfect solution.

Ultimately, sample selection is a key challenge that affects nearly all seroprevalence studies—if the sample differs from the wider population in ways that affect the likelihood of exposure to the virus, then the estimated seroprevalence may be unreliable.

### 3.3. Uncertainty from Sampling

No matter how carefully we choose our sample, it is unlikely that the prevalence of past infection among those sampled will be exactly the same as that of the wider population—some variation is inevitable due to random chance. Typically, this variation is measured via the use of interval estimates (e.g., confidence interval and credible intervals). While many readers will be familiar with these ideas, there is evidence suggesting that they are widely misunderstood, even by experts [48]. For this reason, we will give a brief and nontechnical introduction to interval estimates in the context of seroprevalence studies, clarifying which sources of uncertainty are captured by these estimates, and which are not. Detailed discussions on the interpretation (and misinterpretation) of these ideas can be found elsewhere [49,50].

As a motivating example, imagine that the true prevalence of past SARS-CoV-2 infection in the population is 5%, and that we randomly selected a sample of 100 people from the population. We would not be hugely surprised if the number of people in the sample previously infected were not exactly 5. It is easy to imagine such a sample containing 1, 3, 10, or even 12 previously infected people, simply due to chance. Thus in a seroprevalence study of only 100 people, we could quite easily obtain very inaccurate estimates of say 1% or 12%, from which we could draw misleading conclusions. In a much larger sample of say 100,000 people, we still would not expect exactly 5000 (5%) to have been previously infected. However, finding as few as 1000 or as many as 12,000 would be extremely unlikely. Thus, as the sample size increases, estimates that are very wrong due to chance become less likely, and we can be more confident that our results are close to the true value—provided the sample has been selected appropriately. In fact, based on the sample size, we can measure uncertainty due to random variation. We can calculate how far away the estimate (i.e., the prevalence in the sample) could reasonably be from the true value (the prevalence in the wider population), subject to an acceptable level of uncertainty.

To describe this, results of seroprevalence studies consist of two elements:

- A point estimate, i.e., a single “central” or “most likely” value for the percentage of people who are estimated to have been previously infected.
- An interval estimate, or a range of values surrounding the point estimate. This could be called a confidence/credible/uncertainty interval. They represent the range in which we expect the “true” value of prevalence to lie, with a reasonable degree of certainty, and we consider values outside this interval to be unlikely. Thus, a wider interval suggests a greater degree of uncertainty compared with a narrow one.

**Figure 3:**
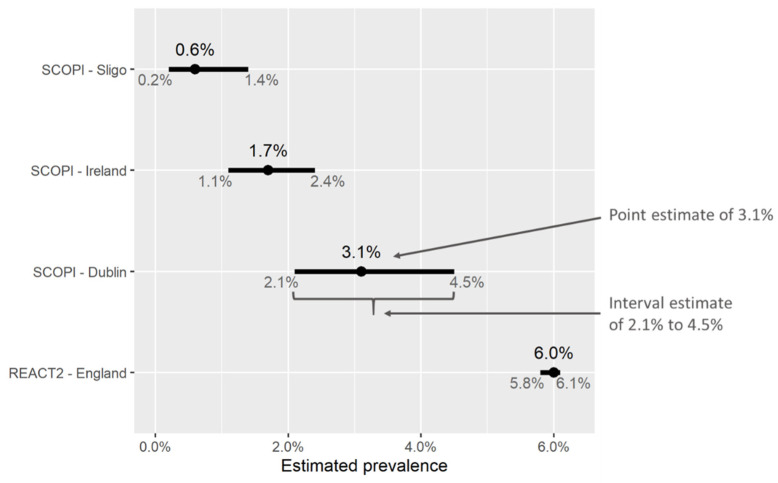
Point and interval estimates of seroprevalence from two studies, SCOPI [24] in Ireland and REACT2 [51] in England. Based on a sample of 913 people in Dublin, the SCOPI study estimated seroprevalence at 3.1%. The corresponding interval estimate indicates that the true value of seroprevalence in the wider population in Dublin was likely to lie between 2.1% and 4.5%. By contrast, the REACT2 study in England enrolled a large number of participants (ca. 100k), and thus the corresponding confidence interval for seroprevalence is narrow—greater sample sizes give more precise estimates (provided they are indeed representative of the population).

Figure 3 shows some examples of point and interval estimates of prevalence obtained from two different studies, SCOPI [24] in Ireland and REACT2 [51] in England. These intervals account for uncertainty due to random variation, such as the difference in prevalence between the sample measured and the wider population. The main factor influencing the width of an interval estimate is the sample size; studies enrolling larger numbers of people will result in less uncertainty, i.e., narrower intervals. As such, analyzing and comparing subgroups of the study population usually results in wider confidence intervals due to smaller sample sizes. In cases where prevalence estimates are adjusted (for example, so as to ensure that the adjusted sample better represents the target population) the width of interval estimates will also be affected (for example, [43,52]).

When results are reported, emphasis is often placed on the point estimate, and the width of the corresponding interval estimate is ignored; media coverage of these studies typically reflects this. This is problematic particularly when intervals are wide, as other values of prevalence (other than that given by the point estimate) may be likely. For example, in Figure 3, the upper end of the interval estimate for seroprevalence in Sligo (1.4%) is seven times higher than that of the lower estimate (0.2%); we can conclude that seroprevalence in Sligo was likely quite low (below 1.5%) but not zero, though we cannot be much more certain than this. Similarly, the PRECISE study [25] estimates seroprevalence for a number of subgroups of hospital workers in Ireland (grouped by age, sex, ethnicity, location, etc.)—while the point estimates vary considerably between groups, sample sizes are small and confidence intervals often overlap. This indicates that some of these observed differences may have occurred by chance, which should be taken into account if these estimates are used to inform infection-control strategies in hospitals or vaccine prioritization. Thus, it is always better to focus on the “plausible range” of prevalence values implied by a study, rather than focusing on the point estimate alone.

Finally, it is equally important to note that not all sources of uncertainty will be captured in these interval estimates. For example, if the sample of people that we have recruited is not representative of the wider population (and we do not adjust it accordingly), then the true population prevalence could still be quite far outside the interval. In general, the validity of any interval estimate depends on the study methodology: design, recruitment, collection, and analysis. Since under real-world conditions, none of these are likely to be perfect, it is prudent to view interval estimates as “best-case scenario” estimates of uncertainty. The extent of additional uncertainty not captured by these intervals is often a matter of qualitative judgment and should not be overlooked.

### 3.4. The Limitations of Antibody Tests

**Figure 4:**
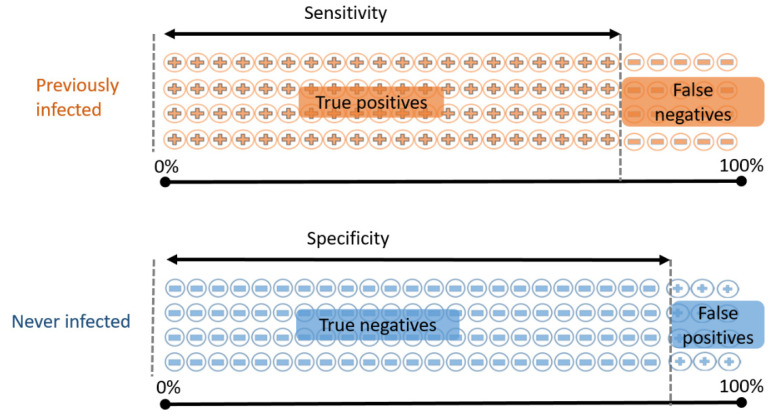
Sensitivity and specificity of a SARS-CoV-2 antibody test. In a sample of previously infected people, some will correctly test positive (true positives) while others will incorrectly test negative (false negatives)—sensitivity refers to the proportion of previously infected people who correctly test positive. Similarly, never-infected people may correctly test negative (true negatives), or incorrectly test positive (false positives)—specificity is the proportion of never-infected people who correctly test negative.

Like many medical tests, no SARS-CoV-2 antibody test is 100% accurate. There are two different ways in which we want an antibody test to be accurate—those who have been previously infected should test positive, and those who have never been infected should test negative. As such, test accuracy is usually described in terms of two separate measurements (Figure 4):

- Sensitivity: the proportion of previously infected people who will correctly test positive;
- Specificity: the proportion of never-infected people who will correctly test negative.

A “perfect” test would have both sensitivity and specificity of 100%. However, false positive and false negative results are inevitable when measuring a binary outcome (such as presence versus absence of SARS-CoV-2 antibodies), arising, for example, from unpredictable variations in biological or chemical reactions to blood samples from different individuals. When such tests are designed there is often a tradeoff to be made, in that sensitivity can be increased at the expense of decreased specificity, or vice versa [53]. Separately, there are practical challenges that may conceivably also affect test performance: sample collection and storage, contamination, laboratory conditions and practices, “cross-reactivity” of other types of antibody with the target antigen, and many others.

To illustrate these concepts at a cohort level, we consider a hypothetical SARS-CoV-2 antibody test that has 80% sensitivity and 94% specificity. This means that

- If we test 100 people who have all previously been infected, we expect 80 of them to test positive.
- If we test 100 people who have never been infected, we expect 94 of them to test negative.

**Figure 5:**
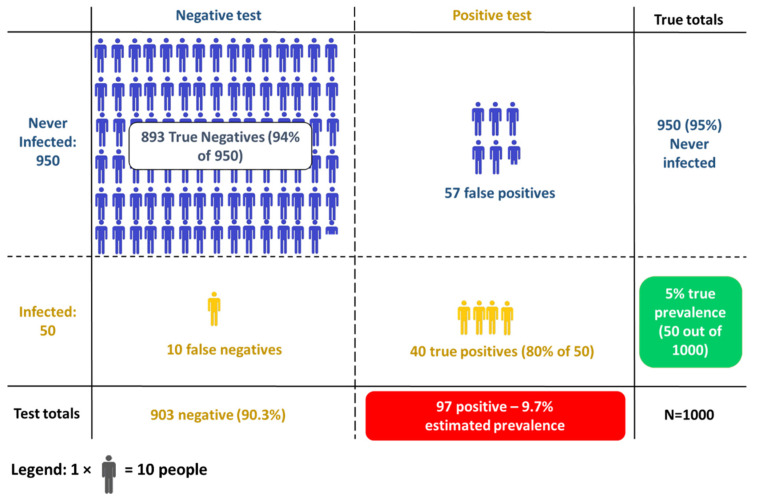
The outcome of testing 1000 people, 50 of whom have previously been infected, using an antibody test with 80% sensitivity and 94% specificity. The true prevalence is thus 5% (50 out of 1000), while the apparent prevalence, i.e., the proportion of tests that give a positive result, is 9.7%.

To see why this matters we imagine an antibody study of 1000 people, 50 of whom (5%) have previously been infected with SARS-CoV-2. We use this same test with 80% sensitivity and 94% specificity. The results are broken down in Figure 5. One aspect jumps out—there are more false positives than true positives, and estimated prevalence (9.7%) is considerably higher than true prevalence (5%). This occurs because:

- Of the 50 people who have been infected, 40 correctly test positive (80% sensitivity)
- Of the 950 people who have not been infected, 893 correctly test negative (94% specificity), and thus 57 test positive.

Adding these together we see that 97 people in total test positive, while the study contained 50 true cases—thus, our estimate is almost twice the true value.

The magnitude of how far the estimated seroprevalence will be from the true prevalence depends not only on the test, but also on the true prevalence in the population. Table 1 shows the results obtained using the same test as before on a sample of 1000 people. If the true prevalence is 2%, a study like this gives an estimated prevalence of 7.5%, which is almost four times higher—an overestimation of this magnitude can lead us to very misleading conclusions. On the other hand, if the true prevalence is 20%, the test performs much better, with an estimate of 20.8% prevalence, which may well be “close enough” for many purposes. These examples show that test accuracy can potentially make a big difference to the estimated prevalence numbers and lead to very misleading results in some cases; when true underlying prevalence is low, tests with high specificity are needed to obtain useful estimates. In other settings sensitivity may be a greater concern; for example, in the PRECISE study of healthcare workers, a comparatively high-prevalence population, obtaining high sensitivity was prioritized [25].

Estimates of the sensitivity of commercially available antibody test kits vary considerably, with many exhibiting sensitivity below 80% [18,54,55]. Specificity is generally higher, typically above 95% and in many cases above 98%; however, when true seroprevalence is low this can still result in many false positives. By comparison when testing for active infection via RT-pCR, specificity is generally understood to be very high (>99%), while estimates of sensitivity vary (being highly dependent on swabbing location, technique, and timing relative to infection) but are almost certainly lower than 95% in clinical practice (possibly much lower) [13,14,15]. Thus, false positive RT-pCR tests are relatively rare, with false negatives more common, while the corresponding rates for antibody tests depend on the performance of the test kit used and the true underlying population prevalence.

The accuracy of antibody tests was central to the debate surrounding the Santa Clara seroprevalence study (specifically concerning the potential overestimation of infection numbers due to false positives), and has arisen in numerous other studies [7,8,56]. These issues should not be overlooked when studying the spread of infection and developing related policy; for example, the potential impact of imperfect test accuracy on estimated seroprevalence should be explored, particularly in cases where the results are surprising. The antibody test kit used in a seroprevalence study should be chosen with these concerns in mind (taking into account expected prevalence in the population); otherwise, the results obtained may be of limited value.

### 3.5. Challenges When Correcting for Imperfect Test Performance

When faced with imperfect tests in seroprevalence studies, we have two options:

- Accept that this is a limitation of the study, and that the true prevalence might therefore differ from what was estimated in the study. In this case, we are estimating the proportion of the population who would test positive, using the same test.
- Try to adjust or correct the results to account for imperfect test accuracy (i.e., attempt to subtract the false positives and add the false negatives). In this case, we are estimating the proportion of the population who have SARS-CoV-2 antibodies in their blood.

For example, in a study [34] of healthy blood donors in England, unadjusted seroprevalence at the end of September 2020 was estimated at 5.7% (with a confidence interval of 5.2% to 6.3%), while adjusting for sensitivity and specificity gave an estimate of 6.1% (confidence interval 5.4% to 6.8%). For most users of seroprevalence studies, the second approach seems preferable, as it aims to adjust for the problems caused by imperfect test performance and estimate the true quantity of interest—the prevalence of antibodies. If only unadjusted results are reported, the reader must assess the extent to which they may be affected by imperfect test performance.

Correcting or adjusting “apparent prevalence”, that is, converting the proportion of positive tests to an estimate of “true” seroprevalence, is a simple calculation once test sensitivity and specificity are known [57]. However, in practice this is rarely the case, as these parameters also need to be estimated using appropriately designed studies (often called validation studies), which typically involve testing a number of blood samples with “known” past COVID-19 infection status, to estimate test sensitivity and specificity. Validation studies also come with all the challenges of population and sample selection, and the effects of random variation, which gives rise to uncertainty in the resulting estimates of sensitivity and specificity. As these parameters are used when calculating (test-adjusted) seroprevalence, the latter is also affected by this uncertainty. This should be reflected in the associated (confidence/credible) interval estimates for seroprevalence (though this may be overlooked in some studies); different approaches are discussed in [57,58,59,60,61]. When done correctly, adjustments for test accuracy usually result in wider confidence intervals, compared with the unadjusted results, reflecting the additional unknowns (sensitivity and specificity) in the calculation. It is important to note that while adjusting for test performance results in wider interval estimates, the adjustment itself does not increase uncertainty—these intervals merely acknowledge uncertainty that is already present.

**Figure 6:**
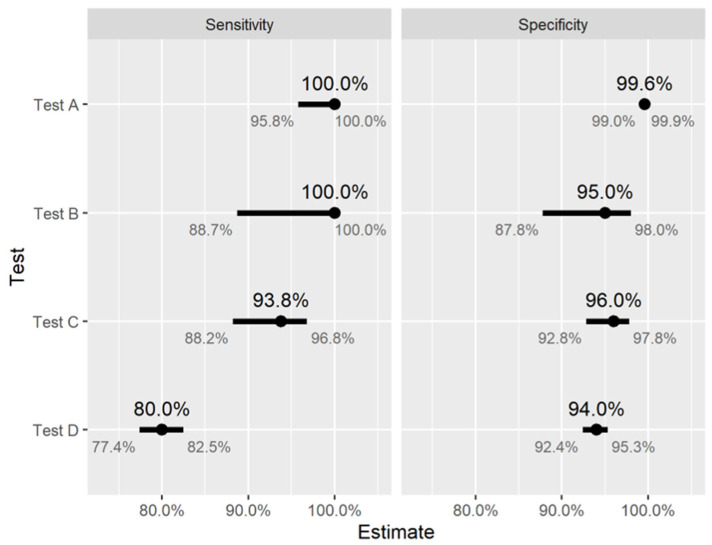
Estimated sensitivity and specificity of four different antibody tests. Tests A, B, and C are based on data presented on the US Food and Drug Administration website (for three different commercially available tests), Test D is for illustrative purposes. Estimated sensitivity and specificity for Test A are both high, with a high degree of certainty (i.e., narrow interval estimates). For Tests B and C, estimated sensitivity and specificity are more uncertain, while for Test D these values are definitely lower.

Figure 6 shows estimated sensitivity and specificity, together with associated confidence intervals for three commercially available tests, taken from the US Food and Drug Administration (FDA) website [62] (Tests A, B and C correspond to Abbott Architect SARS-CoV-2 IgG, Cellex qSARS-CoV-2 IgG/IgM Rapid Test, and Megna Health Rapid COVID-19 IgM/IgG Combo Test Kit, respectively). One possible reason for the differences in test performance is the fact that Test A is laboratory-based chemiluminescent microparticle immunoassay, while B and C are point-of-care LFIAs (which are generally regarded as less accurate [18,55]). Moreover, the validation studies used to obtain these estimates were carried out separately, using different samples and study designs, which may also explain some of the differences in observed test performance. A recent review of studies evaluating antibody tests noted that many of them were of low quality [55], and therefore it is quite possible that the intervals in Figure 6 actually underestimate uncertainty, and any comparison of these estimates between tests should be interpreted with caution. To account for the possibility of test performance being lower, we have also included a fourth (hypothetical) test with lower sensitivity and specificity (Test D), for illustrative purposes.

**Figure 7:**
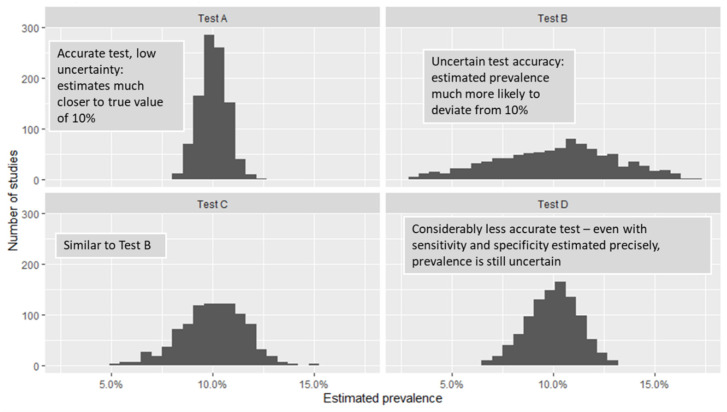
Estimates of seroprevalence from 10,000 simulated studies, using different antibody tests and adjusting results for imperfect sensitivity and specificity. Each simulation selects a random sample of 2500 participants from a population with an overall prevalence of 10%. The extent to which estimated prevalence differs from true prevalence on average depends on the test used—lower and/or more uncertain values of sensitivity and specificity result in more uncertain estimates of prevalence.

Studies carried out using highly accurate tests (i.e., those with point estimates of sensitivity and specificity near 100%, and narrow interval estimates for these values) will give the most precise estimates of seroprevalence in the population, compared to those using antibody tests with low or uncertain estimates of sensitivity and specificity. These factors limit how precisely we can estimate true seroprevalence in the population and can vary considerably between tests. Figure 7 illustrates how this occurs by plotting the results of 10,000 “simulated” (i.e., random, computer-generated) studies carried out using different antibody tests. In each simulation, we pick a random sample of size 2500 from a very large population, in which the true seroprevalence is 10%, and test them using Tests A, B, C, and D from Figure 6. We then “correct” the results to account for sensitivity and specificity and estimate the seroprevalence. The prevalence of antibodies in each sample differs randomly from that of the true population, as do estimated sensitivity and specificity, to account for what would happen in real-life.

The graphs show the distribution or “spread” of the results from these simulated studies and illustrate how much these typically differ from the true prevalence of 10%. In each plot, the horizontal axis represents the estimated prevalence, after adjusting for test sensitivity and specificity. The height of the bars represents the number of simulated studies resulting in that particular estimate; thus, if the bars are clustered close together, the corresponding test is likely to give very precise estimates of seroprevalence that are close to the true value of 10%. These estimates are centered around the true value of 10% no matter which test we use. However, those for Test A are typically much closer to the true value than any of the other tests; this reflects the fact that Test A is both more accurate (i.e., sensitivity and specificity are closer to 100%), and also that we are more certain about these values (see Figure 6). Note that with Test D, even though we have very precise estimates of sensitivity and specificity, these values are quite low; as a result, estimates of seroprevalence are quite variable. A consequence of this is that in many situations, tests with low specificity (e.g., less than or equal to 100% prevalence) may produce estimates of seroprevalence that are too uncertain to be of practical use (even when adjustment is carried out correctly), e.g., [60].

The uncertainty just described can be incorporated into interval estimates for seroprevalence, though some studies may fail to do so correctly. However, there is potentially much more uncertainty than the simulations in Figure 7 suggest. Indeed, the confidence intervals in Figure 6 only capture uncertainty arising from random chance, and simply reflect the sample size of the validation study—the width of the intervals essentially describes how much variation in estimated sensitivity and specificity would be expected if the validation studies were repeated using different random samples selected from the same population. If these data are to be used to adjust the results of a seroprevalence study, other factors that might cause test performance to differ from what was observed in the validation studies must be considered:

- Population heterogeneity: factors such as age, sex, pre-existing conditions, and others may affect antibody response to COVID-19 infection [63], and thus influence test performance. Exposure to other circulating coronavirus may give rise to cross-reactive antibodies [64], which could affect test specificity; thus, geographic location may also be a meaningful source of heterogeneity.
- Patient history: disease severity and time since infection are likely to affect antibody levels [63]. If these factors differ between validation and seroprevalence studies, then test sensitivity may also differ.
- Sample collection, storage, and laboratory processing standards may affect test performance and also differ between studies.

Ideally, adjusting for test performance would require reliable estimates of test sensitivity and specificity in the same population (or a very similar one) under the same conditions. Such information may not be available to study investigators in many cases; thus, the validity of any such adjustment must be assessed in light of these uncertainties. If unadjusted estimates are available, it may be possible to reanalyze the data using other plausible estimates of sensitivity and specificity (see [60] for an example of such an analysis in the context of the Santa Clara study [7]).

In summary, while we can correct seroprevalence results to account for test accuracy, doing so involves incorporating additional uncertainties, some of which are captured via wider confidence intervals (i.e., less precise estimates). However, the quality and appropriateness of the information we use (i.e., estimates of sensitivity and specificity) is another source of uncertainty that cannot be easily quantified and will not generally be present in these interval estimates. When evaluating these studies, it is therefore important to confirm that appropriate statistical methods have been used to adjust for test performance, but also to assess whether or not the estimates of sensitivity and specificity used are likely to be applicable to the study population.

### 3.6. Antibodies, Immunity, and Vaccines

In many studies, the presence of specific antibodies is assumed to be a reasonable marker of previous SARS-CoV-2 infection. However, there are questions about the long-term persistence of antibodies in the blood of recovered COVID-19 patients, with some studies reporting that antibodies decrease to undetectable levels over time [65,66] (particularly among mild and asymptomatic patients), though other studies have shown the opposite [20,67]. As such, seroprevalence surveys may do a better job at identifying recent infections than those that occurred earlier in time but will inevitably “miss” an unknown proportion of cases. This proportion is likely to vary depending on the dynamics of infections over time in the population of interest, which can make the impact difficult to assess in any given study.

Similarly, the relationship between antibodies and immunity remains unclear—it is not known whether or not the presence of SARS-CoV-2 antibodies indicates immunity, nor whether the absence of detectable antibodies is evidence of no immunity. While confirmed reinfections have been documented [68,69], there is emerging evidence that supports the hypothesis that antibodies provide substantial (but not complete) protection from reinfection over the medium-term [70,71]. Conversely, we have previously discussed the example of Manaus, Brazil, experiencing a resurgence of COVID-19 cases [10], despite evidence of possible herd immunity being attained [9]. In addition to the limitations of sampling blood donors, the possible role of waning immunity or antibody-evading variants causing reinfections have been discussed [10]. Of note, the estimate of a 76% historic infection rate was obtained after adjustment for declining antibody levels over time (estimated seroprevalence was 52.5% in June and only 25.8% by October), indicating that some loss of immunity may have taken place. Either way, this example highlights the risks of assuming that herd immunity has been reached based upon seroprevalence studies, particularly if this information will be used to inform policy decisions such as lifting NPIs. Ultimately, while researchers and policymakers may decide to use seroprevalence studies to estimate the extent of population immunity to COVID-19, doing so requires making additional assumptions that we cannot yet verify.

With the ongoing rollout of mass vaccination campaigns in many countries, a new challenge emerges: the presence of SARS-CoV-2 antibodies in an individual’s blood could indicate vaccination, previous infection, or both. This is acknowledged in the more recent serosurveillance reports published by the UK Office for National Statistics, for example [72]. This may not be a problem if the goal of the study is to estimate current levels of population immunity (subject to the assumptions discussed previously). However, for most other purposes it is likely that investigators will wish to distinguish between vaccination and past infection. As such, seroprevalence surveys carried out using the currently available antibody testing kits may no longer be a reliable method of estimating the prevalence of previous infection in a population, if vaccination has been carried out in that population (depending on the type of vaccine received and on the target antigen of the test kit [73]).

## 4. Conclusions

Estimating the number of previous SARS-CoV-2 infections in a given population is important for understanding the nature of the disease and its transmission, and the effectiveness of strategies used to control it. Seroprevalence studies are a well-established approach to doing this, although there are many challenges in estimating this number accurately, and any such estimate will be uncertain (though to what extent can vary considerably between studies). While the main sources of this uncertainty outlined in this paper are generally well understood in the statistics and epidemiology communities, the nature of the COVID-19 pandemic necessitates that the results of these studies (and their limitations) be understood by those with varying levels of statistical knowledge. The issues discussed here are not unique to seroprevalence studies, and can arise in any scientific study where we recruit a sample of people in order to estimate the prevalence of some factor of interest in a wider population, using an imperfect test. Generally, interval estimates such as confidence intervals allow us to measure the likely effect of random variation on the results; however, they rarely capture all of the uncertainty that arises when trying to use these results to better understand the disease or to make decisions. It is therefore important to be aware of other sources of uncertainty: issues around restricted populations, nonrepresentative samples, and importantly, what exactly is being measured and how (e.g., in our case, uncertainty around test accuracy, and limited knowledge about antibodies in the long term). Awareness of the issues discussed in this article allows the quality of a given study to be assessed and to better assess the certainty and the limitations of the results obtained.

Despite these limitations, well-designed seroprevalence studies are still usually the best approach to estimate previous infections and current levels of immunity in the target populations. However, the estimates that they provide should be interpreted in the context of other external information, such as confirmed cases, deaths, and infectious disease models, to better understand the disease.

## Figures and Tables

**Table 1:** Two examples of estimated seroprevalence using a test with sensitivity 80% and specificity 94%.

	Example 1: True Prevalence 2%			Example 2: True Prevalence 20%		
	Negative	Positive	Total	Negative	Positive	Total
Never infected	921	59	980	752	48	800
Previously Infected	4	16	20 (2% true prevalence)	40	160	200 (20% true prevalence)
Total	925	75 (7.5% estimated prevalence)	1000	792	208 (20.8% estimated prevalence)	1000

## Data Availability

Not applicable.
